# Colorectal Cancer in Northwestern Iran

**DOI:** 10.5402/2012/968560

**Published:** 2012-06-12

**Authors:** Rahim Mahmodlou, Payvand Mohammadi, Nariman Sepehrvand

**Affiliations:** ^1^Department of Surgery, Imam Khomeini Training Hospital, Urmia University of Medical Sciences, Urmia, Iran; ^2^Department of Internal Medicine, University of Medical Sciences, Urmia, Iran; ^3^Students' Research Committee, Urmia University of Medical Sciences, Urmia, Iran

## Abstract

*Background*. Colorectal cancer is the third most prevalent cancer worldwide, which is less common in the Middle East; its prevalence demonstrated to be 7 persons per 100,000 populations in Iran. In this study, we aimed to investigate the clinicopathologic features of CRC patients in West Azerbaijan province of Iran. *Methods*. In this crosssectional study, 546 patients who underwent surgical procedures with the pathologic diagnosis of colorectal cancer in both academic and private hospitals of Urmia were enrolled. *Results*. The mean age at diagnosis was 55.2 ± 11.5 years. 23% had an age lower than forty years old. Rectal bleeding (26%), abdominal pain (25%), and large bowel obstruction (23%) were three most common presenting symptoms. 26% of patients presented with acute abdomen. 95% of tumors were adenocarcinoma, 4% were lymphoma, and 1% was other rare tumors. Regarding the stage of cancer according to TNM staging system, 6% of patients were in stage I, followed by 37% in stage II, 33% in stage III, and 24% in stage IV. In 44.5% of patients, the tumor was located in rectum. *Conclusion*. In west Azerbaijan province of Iran, patients with colorectal cancer present in younger age and more advanced stages in comparison with the developed countries.

## 1. Introduction

Colorectal cancer (CRC) is the third most common cause of cancer diagnosed in the United States and the second leading cause of cancer death [1]. According to the reports, every 9 minutes, someone dies from CRC [2]. CRC is less common in the Middle East [3]. Also, in WHO EMRO region, it has the 3rd place among the most common cancers (3rd place after breast and cervical cancer in women and 5th place after lung, bladder, stomach cancer, and non-Hodgkin lymphoma) [4]. 

Colorectal cancer is the 3rd and the 4th most commonly diagnosed cancer, respectively, in Iranian men and women [5]. The prevalence of CRC demonstrated to be 7 persons per 100,000 population in Iran [6]. 5000 new Iranian cases of CRC reported each year [5]. Although there is some information about the situation of CRC, from different regions of Iran, no specific data were available regarding the prevalence and clinicopathologic characteristics of CRC in West Azerbaijan province located in the northwestern Iran. In this study, we aimed to investigate the clinicopathologic features of CRC patients in West Azerbaijan province of Iran. 

## 2. Material and Methods

**Figure 1:**
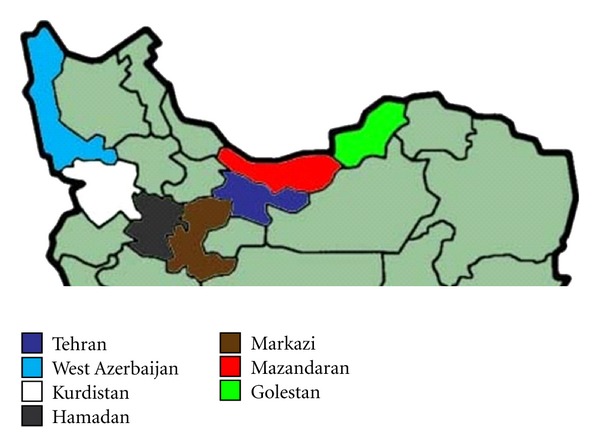
The location of West Azerbaijan province (Our study) in the northwest of Iran, compared to the place of previous reports from Iran (the Tehran, Markazi, Hamadan, Kurdistan, Golestan, and Mazandaran provinces).

This hospital-based cross-sectional descriptive study was approved by the Scientific and Ethical Review Board of Urmia University of Medical Sciences. It was conducted at academic training and private hospitals of Urmia, the capital of West-Azerbaijan province. The location of West Azerbaijan province in the northwest of Iran is depicted in Figure 1. This figure also demonstrated the Tehran, Markazi, Hamadan, Kurdistan, Golestan, and Mazandaran provinces from which previous Iranian studies regarding the epidemiology of colorectal cancer were reported. 

546 patients who underwent surgical procedures with the pathologic diagnosis of colorectal cancer within 8 years (2001–2008) were enrolled in this study. The following data were obtained: age at diagnosis, gender, presenting symptoms (including abdominal pain, change in bowel habit, hematochezia or melena, weakness, anemia, and weight loss), anatomical site of tumor, and pathological type of tumor (grade and stage of tumor). 

In this study, the TNM (tumor, lymph nodes, metastasis) staging system was used for determining the severity of disease and the local or distant extent of disease spread. The TNM staging system of the American Joint Committee on Cancer (AJCC) is the preferred staging system for CRC. 

Statistical analysis was performed using SPSS software ver16 package. Frequencies were provided using descriptive statistics. 

## 3. Results

A total of 546 patients were enrolled in this study. 306 patients (56%) were men, and 240 (44%) were women. The mean age at diagnosis was 55.2 ± 11.5 years old. Among patients, 33.6% had an age lower than fifty, 43.4% were between 50 and 69 years, and 22.9% had an age higher than 70 years old. 23% of patients had an age lower than forty years old.

The presenting symptoms were rectal bleeding (26%), abdominal pain (25%), large bowel obstruction (23%), change in bowel habits (14%), weakness and anemia (5%), abdominal mass (4%), and bowel perforation and peritonitis (3%). Overall, in 26% of them, the patients were presented with acute abdomen due to bowel obstruction, perforation, or the like.

Regarding the histological diagnosis, 95% of tumors were adenocarcinoma, 4% were lymphoma, and 1% was other rare tumors. About the grade of tumor, 64% had well-differentiated tumors, followed by moderately and poorly differentiated tumors, respectively, in 30% and 6% of patients.

Regarding the stage of cancer according to TNM staging system, 6% of patients were in stage I, followed by 37% in stage II, 33% in stage III, and 24% in stage IV. 

The tumor was located in rectum in 244 patients (44.5%), right colon in 192 patients (35%), and left colon in 110 cases (20%).

## 4. Discussion

The prevalence of cancer in Iran is much higher than the universal average (160 per 100,000 Iranian population compared to 66.8 and 73.9 per 100,000 for women and men worldwide). This rate is 201 cases per each 100,000 population for the West Azerbaijan province, which is significantly higher than other parts of the country. West Azerbaijan has the forth place after Guilan (223.4/100,000), Isfahan (218/100,000), and Yazd (217.4/100,000) provinces [7]. Colorectal cancer is the third most common cancer worldwide. Esna-Ashari et al. investigated the prevalence of colorectal cancer in Iran according to the survival data in a 5-year period (2001–2005) and estimated the prevalence in the 1st year, 2nd-3rd, and 4th-5th years to be 4156, 5715, and 4283 cases. The cumulative prevalence in these 5 years was 13954 cases, but despite the high rate of new cases, the point prevalence was not considerably changed in this period [8]. According to the study of Moghimi-Dehkordi et al., the Kaplan-Meier indicated the 1-, 3-, 5-, 10-, 15-year survival rates of Iranian CRC patients to be 91.1, 73.1, 61, 54.9, 25, and 9 percent, respectively [9].

There is a paucity of data regarding the colorectal cancer from northwestern provinces of Iran. Some important characteristics demonstrated in our findings were listed as follows.

**Age Distribution**

The mean age of our patients was 55.2 ± 11.5 years old. The age distribution of CRC patients, mentioned in different Iranian studies varies, from 51 years in Kurdistan province to almost 60 in Tehran. CRC patients in central provinces such as Tehran (59.7 years) [10], Hamedan (58.6 years) [11], and Markazi (56.2 years) [12] seem to have higher age than patients from northern and north western provinces of the country (Gorgan: 52–56 years [13, 14], Mazandaran: 52 [5], West Azerbaijan: 55, and Kurdistan: 51 years old [6]). Only the study of Jalali et al. from Tehran province reported a low mean age such as 51 years old, which could be due to the referral nature of the Imam-Khomeini General Hospital, in which the mentioned study was conducted [15]. The patients, who referred to Imam Khomeini Hospital of Tehran (the capital of Iran) from other provinces, may carry the characteristics of CRC in their region to the capital. Although mean age is generally in concordance with other studies (in a range between 51 and 59 years old), the ratio of patients with an age under forty was demonstrated to be 23% in our province, which is against many other studies. For example in the study of Li et al. in China, 10% of their patients have an age under 40 [16]. Of course our findings are similar to the findings of Jalali et al. (26%) in Tehran [15]. Considering the fact that colorectal cancer in younger ages may be more due to some probable genetic or familial factors, the genetic and familial background of the CRC patients in our province should be investigated in further studies. Also the more advanced cancer stage in the patients aged below 50 years needs to make the treating physicians more aware of the fact that CRC can occur even at this age [17]. Of course some studies demonstrated that, in younger ages, the cancer is presented with different features (anatomical site, stage of tumor, etc.), but young age by itself was not found to impact patient outcome [16, 18].

**Presenting Sign and Symptom**

In this study, 26% of subjects were presented with acute abdomen because of bowel obstruction or perforation, which is against the findings of others that reported acute and subacute presentation in less than 10% of their patients. It is important because subacute presentations make the appropriate treatment impossible [19, 20]. Delay in diagnosis may be due to overlooking the alarming sign and symptoms by both the patients and physicians. The patient may neglect the alarming signs, and, on other hand, the physician may attribute the patient's symptoms to other benign anorectal diseases. The evidence is the frequency of patients that underwent hemorrhoidectomy several months prior to definitive diagnosis of colorectal cancer. This finding suggests the need for implementing an extensive educational program on general population about the importance of alarming sign and symptoms including changes in bowel habits.

**Pathology**

In our study, the pathology of tumor was adenocarcinoma in 95%, lymphoma in 4%, and rare tumors in 1% which is similar to several other reports [5, 6, 11, 12, 14, 21–23].

**Stage of Tumor**

Unfortunately 57% of our patients were presented in 3rd or 4th stage. This is in accordance with other reports from Iran [15], but this is in contrast with reports from other parts of the world (30% for the 3rd and 4th stage in the study of Liu et al. in China) [19]. This deplorable finding as mentioned previously can be attributed to overlooking the alarming sign and symptoms by the patients and/or the physicians, and mainly due to the lack of an appropriate screening program for CRC in our province, as well as country.

**Site of Tumor**

The most prevalent site of tumor was rectum (almost 45%) in our study, unlike in the United States and in concordance with many other studies from this region [3]. The 65% of tumors were located after the splenic flexure. This proposes the applicability of colonoscopy in screening CRC in west Azerbaijan and other parts of Iran.

The increasing incidence of right-sided CRC has been noted in some developed countries; thus, they had to change their screening methodology and to consider other measures such as CT colonography or stool DNA tests beside the more invasive colonoscopy method [1, 17]. 

According to the literature, survival improves substantially if the colorectal cancer is diagnosed while it is still localized [2]. There are several evidences supporting the screening of individuals over the age of 50 years to detect and prevent colorectal cancer [1, 15]. 

Of course it should be noted that, even in countries such as the United States with strong protocols in favor of performing CRC screening programs, every 5 seconds someone who should be screened for CRC is not. And only one third of people who expected to have CRC are screened for it. Unfortunately, 20% of CRC patients who do receive screening demonstrated to be diagnosed in the later, less-treatable stages [2].

Developing a road map for finding the most appropriate screening method and the best age cutoff for performing the CRC screening in Iran is recommended. Reviewing the experience of screening programs in developed countries such as the US could help us in reaching a successful national screening program. The measures should be taken to maximize the compliance of patients and physicians with the mentioned screening program. 

Our limitation in studying the clinicopathologic features of CRC patients in west Azerbaijan province was the population we enrolled that all of them were the patients, which their disease ended to surgery. The patients who die at end stages of cancer without any surgical intervention, or the CRC patients who have not received any surgical treatment yet, were not included in our study. 

## 5. Conclusion

In conclusion, in west Azerbaijan province of Iran, patients with colorectal cancer present in younger age and more advanced stages in comparison with the developed countries, in which almost 60% of CRC cases occur. The authors emphasize on the importance of implementing an appropriate screening program with considering the region-specific characteristics of CRC patients.
